# Parathyroid hemangioma

**DOI:** 10.4322/acr.2021.270

**Published:** 2021-05-25

**Authors:** Odille Mejia, Hisham F. Bahmad, Kei Shing Oh, Juan C. Paramo, Robert Poppiti

**Affiliations:** 1 Mount Sinai Medical Center, Arkadi M. Rywlin M.D. Department of Pathology and Laboratory Medicine, Miami Beach, FL, USA; 2 Mount Sinai Medical Center, Department of General Surgery, Miami Beach, FL, USA; 3 Florida International University, Herbert Wertheim College of Medicine, Miami, FL, USA

**Keywords:** Parathyroid Neoplasms, Hemangioma, Parathyroid Diseases, Adenoma, Case Reports

## Abstract

**Background:**

Hemangiomas are benign neoplasms of capillary proliferation that arise from a developmental anomaly where angioblastic mesenchyme fails to form canals. Most hemangiomas arise in the head and neck region, either superficially in the skin or deeper within endocrine organs such as the parotid gland. Parathyroid hemangiomas, however, are extremely rare, with only five cases previously reported in the literature.

**Case presentation:**

Herein, we present a case of a 68-year-old man with a hemangioma almost completely replacing the right upper parathyroid gland, grossly measuring 1.3 × 1.3 × 1.2 cm and weighing 700 mg, associated with primary hyperparathyroidism.

**Conclusions:**

Parathyroid gland enlargement due to vascular neoplasms such as hemangiomas can mimic, both clinically and radiographically, hyperplasias and/or adenomas. Surgeons need to be aware of the presence of this entity and should consider it in the differential diagnosis of hyperparathyroidism or parathyroid gland enlargement.

## INTRODUCTION

Vascular anomalies encompass a spectrum of disorders that range from a simple “birthmark” to life-threatening entities. The International Society for the Study of Vascular Anomalies (ISSVA) introduced a uniform classification stratifying vascular lesions into vascular malformations (defects in morphogenesis) and proliferative vascular lesions (tumors).^1^ In the head and neck region, vascular lesions include hemangiomas, hemangioendotheliomas, and angiosarcomas, among others.^2^ ^-^ ^4^ Hemangiomas are benign neoplasms of capillary proliferation.^5^ It is hypothesized that these neoplasms arise from a developmental anomaly where angioblastic mesenchyme fails to form canals.^6^ Sixty-five percent of hemangiomas reported arise in the region of the head and neck^7^ with the majority located superficially in the skin,^8^ while others arising deeper within the tongue and the larynx or within endocrine organs such as the parotid gland.^9^ Other endocrine glands that can present vascular malformations include the ovaries, thyroid, and adrenals.^9^ ^-^ ^11^ However, parathyroid hemangiomas are extremely rare, with only five cases previously reported in the literature (Table 1).

**Table 1:** Review of the five previously published cases of parathyroid hemangiomas in literature

Reference	Age/Gender	Medical History	HPTH	Location	Hemangioma
^10^	68/F	Polymyalgia rheumatica, Osteoporosis, Treated hypothyroidism, Hypertension, Primary hyperparathyroidism, and hypercalcemia	Present	Right lower parathyroid	Capillary
	62/M	10-year history of recurrent duodenal ulcer, Renal calculi, Primary hyperparathyroidism	Present	Right lower, left lower, and left upper parathyroid	Cavernous
^12^	53/M	Hypertension, Diabetes mellitus 2, Primary hyperparathyroidism for 8 years	Present	Left lower parathyroid	Capillary
^11^	26/F	1-day history of acute left-sided neck swelling, pain, and pressure	Absent	Left parathyroid	Not specified
^13^	30s/M	Cluster headaches, Migraines	Absent	Retropharynx	Not specified
Current Case	68/M	Hypertension, Subclinical hyperthyroidism presenting as multinodular goiter for 2 years, Primary hyperparathyroidism	Present	Right upper parathyroid	Capillary

F= Female; HPTH= Hyperparathyroidism; M= Male.

While hemangiomas arising in the parathyroid gland are more commonly associated with primary hyperparathyroidism and the diagnosis is almost always made postoperatively, no pertinent medical history of hyperparathyroidism has been reported in two of the five cases published. Merino et al.^10^ first described this entity in two patients in 1996, followed by Svec and Bury^12^ in 2010, Nguyen et al.^11^ in 2011, and Snyder et al.^13^ in 2018.

Here, we are presenting a case of a 68-year-old man with a hemangioma almost completely replacing the right upper parathyroid gland associated with primary hyperparathyroidism. We describe the microscopic features associated with this entity and summarize the other cases reported in world literature. Surgical CAse REports (SCARE) guidelines for reporting case reports were followed.

## CASE REPORT

A 68-year-old man presented to our institution complaining of fatigue with no other symptoms. The patient had a medical history of hypercalcemia and increased parathyroid hormone levels, as well as subclinical hyperthyroidism (multinodular goiter) for two years. The patient had a history of inguinal hernia repair and was allergic to lisinopril. He was hypertensive and was prescribed losartan 100mg (one tablet per day) to control his blood pressure. Family history was significant for lung cancer in his mother.

On physical examination, his neck had a normal range of motion with no masses, hoarseness, or adenopathy. The thyroid gland was normal to palpation. Laboratory tests showed slightly high calcium levels of 11.0 mg/dL (reference range 8.5-10.1 mg/dL), high parathyroid hormone (PTH)-1 level of 1,158.3 pg/mL (reference range 18.4-80.1 pg/mL), and low PTH-2 level of 11.5 pg/mL (reference range 18.4-80.1 pg/mL).

The patient had the following previous imaging workup at another institution:

- Neck ultrasound (U/S) performed two years ago showed a multinodular goiter with the following dominant nodules: right middle lobe hypoechoic nodule (1.5 × 1.3 × 1.3 cm) (Bethesda IV) and left middle lobe heterogeneous nodule with hyperechoic areas (2.4 × 1.5 × 1.9 cm) (Bethesda II). Bilateral fine needle aspirations (FNAs) revealed a diagnosis of Bethesda II (benign) on the left and unremarkable parathyroid on the right;
- 20 months after the first thyroid U/S, a new U/S revealed a right thyroid lower pole, hypoechoic, heterogeneous, solid nodule with punctate calcifications measuring 2.6 ×1.7 × 1.6 cm and a complex, solid, and predominantly cystic, septated nodule in the left lower pole measuring 2.6 × 2.0 × 2.2 cm with peripheral vascularity. The findings were consistent with multinodular goiter;
- Nuclear medicine (NM) parathyroid scan (Single-photon-emission computed tomography (SPECT)) done after the second thyroid U/S showed a hypoattenuating nodule measuring 2.4 × 1.9 × 1.8 cm in the right thyroid lobe compatible with parathyroid adenoma. A complex cystic lesion measuring 2.5 × 2.4 × 2.2 cm was also noted in the posterior aspect of the left thyroid lobe, consistent with a benign thyroid cystic nodule.

**Figure 1:**
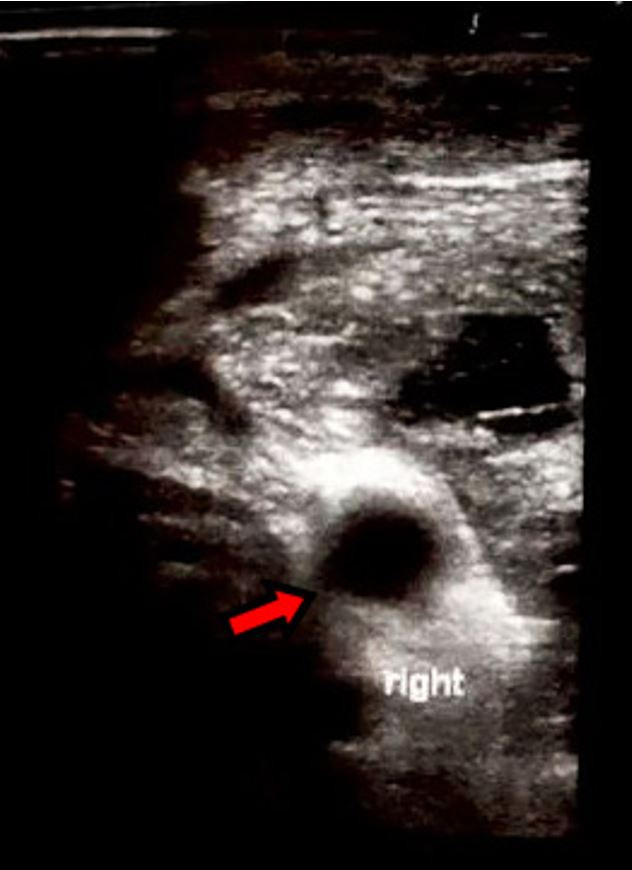
Intraoperative ultrasound revealing a 2 cm heterogeneous right-sided mass consistent with an abnormal right upper parathyroid gland.

The metabolic, nuclear medicine, and imaging workup were consistent with a diagnosis of primary hyperparathyroidism. Based on radiographic studies, an adenoma was presumed to be located in the right inferior parathyroid gland. Minimally invasive radiolocalized parathyroidectomy with laryngeal nerve integrity monitoring was planned. Potential risks and complications were discussed with the patient. Radiolocalization was done, revealing a hot spot on the right side. Intraoperative ultrasound revealed a 2 cm heterogeneous right-sided mass likely consistent with an abnormal right upper parathyroid gland (Figure 1). The latter was carefully dissected and excised. After 15 minutes, intraoperative PTH levels went down to 11 pg/mL, consistent with the resolution of hyperparathyroidism.

**Figure 2:**
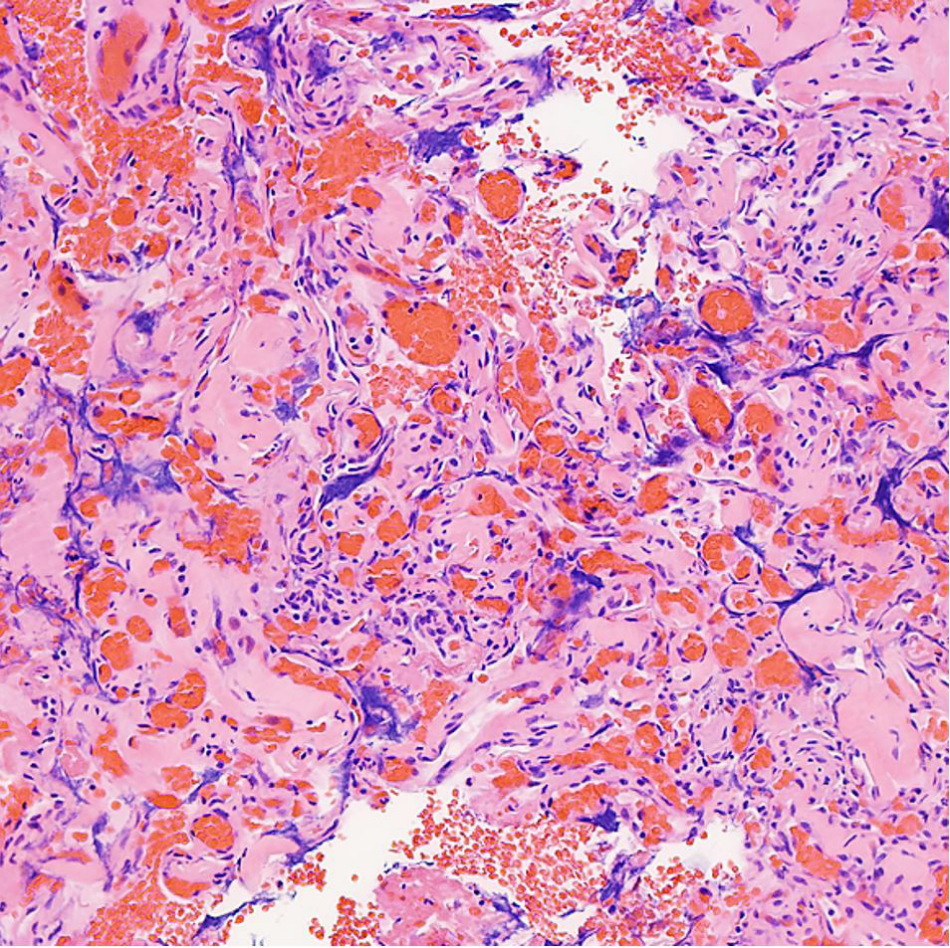
Photomicrographs of the resected parathyroid gland showing multiple endothelium-lined intercommunicating vascular channels filled with blood (capillary hemangioma-like proliferation) without evidence of endothelial atypia or mitotic activity and atrophy of the adjacent tissue (H&E, 20X).

On gross examination, the resected parathyroid measured 1.3 × 1.3 × 1.2 cm and weighed 700 mg (normally, parathyroid glands generally weigh 20-40 mg each; abnormal glands usually weigh >60 mg^14^). It was bisected to reveal a homogenous tan-brown hemorrhagic cut surface. Histopathologic examination demonstrated multiple endothelium-lined anastomosing vascular channels filled with blood without evidence of endothelial atypia or mitotic activity and atrophy of the adjacent parathyroid tissue (Figure 2).

The diagnosis of capillary hemangioma with fibrosis in the milieu of a clinical history of multinodular goiter and primary hyperparathyroidism was made. Immunohistochemical (IHC) stains for vascular markers CD31 and CD34 were performed, highlighting the endothelium lining, and supporting the diagnosis of hemangioma of the parathyroid gland (Figures 33B). Additionally, a minute focus (less than 1mm) of hypercellular parathyroid tissue was identified.

**Figure 3:**
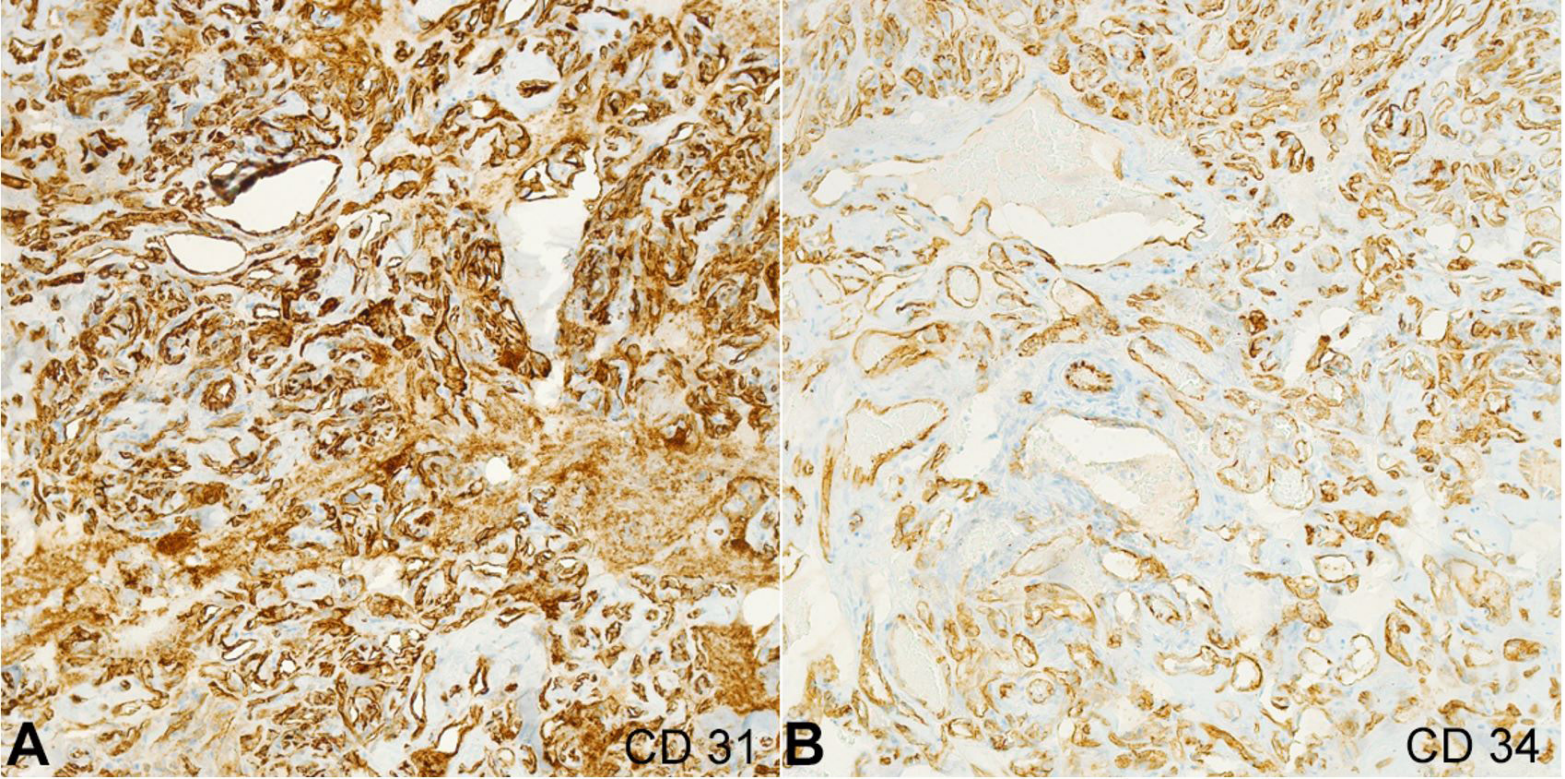
Photomicrographs of the resected parathyroid gland showing immunohistochemical stain for vascular markers, CD31 (A) and CD34 (B) were positive and supported the diagnosis of hemangioma. Slides were stained CD31, and CD34 stains, and images were examined at 20x objective.

The patient was discharged on calcium carbonate 500 mg one tablet by mouth daily. At 3-month follow-up, the patient was doing well without any reported symptoms.

## DISCUSSION

Endocrine organs are highly vascular structures due to their function.^15^ This anatomical characteristic makes them prone to vascular abnormalities, including aberrant proliferation. In the head and neck, hemangiomas of the parotid gland are common.^9^ However, parathyroid hemangiomas are extremely rare and are usually found incidentally in patients diagnosed with primary hyperparathyroidism or adenoma.

A comprehensive search and review of the literature were performed through three databases using MeSH terms and keywords related to “parathyroid” and “hemangioma.” Published articles were retrieved from Medline (OVID), PubMed, and EMBASE since inception. Databases were last searched on January 31, 2021. Results showed five previously published cases of parathyroid hemangiomas,^10^ ^-^ ^13^ with our case being the sixth (Table 1). There is no gender preference for the occurrence of this entity. Three patients were older than 60 years of age. Four patients had a history of primary hyperparathyroidism, including our patient, while no significant medical history of hyperparathyroidism has been reported in two out of the six cases. Unlike the patients in the previous case reports, our patient had a history of multinodular goiter. We hypothesize that the parathyroid hemangioma might be due to a reactive vascular phenomenon secondary to the follicular hyperplasia in the thyroid gland. In a study by Wang et al.,^16^ authors showed that in the absence of TSH hormone, incubation with vascular endothelial growth factor (VEGF) prompted a significant reduction in radioactive nucleoside incorporation of 3H-thymidine into the DNA of cultured rat thyroid FR TL-5 cells. In contrast, incubation with TSH caused a 4-fold increase in 3H-thymidine incorporation. In conclusion, the authors demonstrated that the presence of VEGF and its distribution in thyroid tissues are increased during goiter formation. Therefore, there might be a role for VEGF secreted by the hyperactive thyroid on the parathyroid hemangioma in our patient. This supports the finding that four out of the six parathyroid hemangioma cases reported so far, including our case, have occurred in the context of hyperparathyroidism. In the other two cases where no history of hyperparathyroidism was reported, authors mentioned that in one patient, an organized thrombus was identified on microscopic examination, suggesting that the lesion had been most likely present for a couple of days prior to the acute clinical presentation of a neck mass and, thus, probably representing a primary process.^11^ In the second patient, the parathyroid hemangioma diagnosis was a true incidental finding found on the magnetic resonance imaging (MRI) scan performed to evaluate the patient's history of cluster headaches and migraines, and a right retropharyngeal mass was identified and excised to yield this diagnosis.^13^ Henceforth, we posit that vascular lesions in the parathyroid might be either due to a primary pathologic process in the gland itself or due to a reactive vascular phenomenon secondary to a hyperplastic parathyroid gland and a pathologic process occurring in the vicinity of the parathyroid gland.

The exact pathogenesis of hemangiomas is still unsettled; one theory suggests that they arise from a derangement of angiogenesis, either unregulated angiogenesis or an imbalance of angiogenic and angiostatic factors, including VEGF and fibroblast growth factor (FGF).^11^ On the contrary, Svec and Bury^12^ suggests that there is crosstalk between pro-and anti-angiogenic factors associated with endocrine proliferative lesions such as primary or secondary hyperplasia, adenoma, or carcinoma, with the result being vascular proliferation and acquired hemangioma-like morphology. Yet, the absence of neoplastic and/or hyperplastic cells in the stroma raises questions about a primary pathologic process occurring in the gland and the potential role of secondary angiogenic factors such as vascular endothelial growth factor in parathyroid hemangiomas, as noted by Snyder et al.^13^ Merino et al.^10^ stated that the origin of these lesions is debatable as in other organs. They might represent malformations, hamartomas, or true neoplasms. Some authors described the parathyroid hemangioma cases as being capillary hemangiomas,^10^ ^,^ ^12^ while one case has been reported as cavernous hemangioma.^10^ Indeed, hemangiomas are classified based on the size of the vascular channels as either capillary (small diameter vascular channels) or cavernous (large diameter vascular channels).^17^ ^,^ ^18^ This classification is a pure histopathologic description and does not impose any significant difference in the diagnosis. Lastly, a hypothesis that cannot be ruled out in our present clinical case is the possibility that the vascular alteration in the parathyroid might have been triggered by the trauma due to the fine needle aspiration puncture performed on the thyroid.^19^ ^-^ ^21^

In conclusion, parathyroid gland enlargement due to vascular neoplasms such as hemangioma can mimic, both clinically and radiographically, hyperplasias and/or adenomas. Many of these vascular lesions can occur within a hyperplastic gland. Therefore, surgeons need to be aware of this entity’s presence and should consider it in the differential diagnosis of hyperparathyroidism or parathyroid gland enlargement.

## References

[1] Wassef M, Blei F, Adams D (2015). Vascular anomalies classification: recommendations from the international society for the study of vascular anomalies. Pediatrics.

[2] Brahmbhatt AN, Skalski KA, Bhatt AA (2020). Vascular lesions of the head and neck: an update on classification and imaging review. Insights Imaging.

[3] Nair SC (2018). Vascular anomalies of the head and neck region. J Maxillofac Oral Surg.

[4] Fowell C, Verea Linares C, Jones R, Nishikawa H, Monaghan A (2017). Venous malformations of the head and neck: current concepts in management. Br J Oral Maxillofac Surg.

[5] Dilsiz A, Aydin T, Gursan N (2009). Capillary hemangioma as a rare benign tumor of the oral cavity: a case report. Cases J.

[6] Kumar R, Gupta R, Khullar S, Dasan B, Malhotra A (2000). Thyroid hemangioma: a case report with a review of the literature. Clin Nucl Med.

[7] Shpitzer T, Noyek AM, Witterick I (1997). Noncutaneous cavernous hemangiomas of the head and neck. Am J Otolaryngol.

[8] Kadriyan H, Sulaksana MA, Yudhanto D (2020). Subcutaneous hemangioma on nasal dorsum: a case report. J Med Case Reports.

[9] Kawakami M, Hayashi I, Yoshimura K, Ichihara K, Nishikawa S, Ichihara T (2006). Adult giant hemangioma of the larynx: a case report. Auris Nasus Larynx.

[10] Merino MJ, Chuaqui R, Fernandez P (1996). Parathyroid hemangioma: a report of two cases. Endocr Pathol.

[11] Nguyen PL, Poetker DM, Zambrano E (2011). Parathyroid hemangioma: a case report in proof of its existence. Endocr Pathol.

[12] Svec A, Bury Y (2010). Haemangioma of the parathyroid gland. does it really exist?. Pathol Oncol Res.

[13] Snyder V, Bayrak S, Woodroof J, Kakarala K (2018). A parathyroid hemangioma of the retropharynx. JAMA Otolaryngol Head Neck Surg.

[14] Yeh R, Tay Y-KD, Dercle L, Bandeira L, Parekh MR, Bilezikian JP (2020). A simple formula to estimate parathyroid weight on 4D-CT, predict pathologic weight, and diagnose parathyroid adenoma in patients with primary hyperparathyroidism. AJNR Am J Neuroradiol.

[15] Webb SM, Wägner AM, Lanzer P (2015). Vascular anatomy of the endocrine organs.. Panvascular medicine..

[16] Wang JF, Milosveski V, Schramek C, Fong GH, Becks GP, Hill DJ (1998). Presence and possible role of vascular endothelial growth factor in thyroid cell growth and function. J Endocrinol.

[17] George A, Mani V, Noufal A (2014). Update on the classification of hemangioma. J Oral Maxillofac Pathol.

[18] Donnelly LF, Adams DM, Bisset GS (2000). Vascular malformations and hemangiomas: a practical approach in a multidisciplinary clinic. AJR Am J Roentgenol.

[19] Hovi SI, Kholová I (2017). Vascular proliferation of the thyroid: potential histopathological pitfalls as a consequence of fine needle aspiration. Acta Cytol.

[20] Miao J, Chen S, Li Y, Fu L, Li H (2017). A primary cavernous hemangioma of the thyroid gland: a case report and literature review. Medicine.

[21] Norman J, Politz D, Browarsky I (2007). Diagnostic aspiration of parathyroid adenomas causes severe fibrosis complicating surgery and final histologic diagnosis. Thyroid.

